# Validation of the ABPMpro ambulatory blood pressure monitor in the general population according to AAMI/ESH/ISO Universal Standard (ISO 81060-2:2018)

**DOI:** 10.1097/MBP.0000000000000640

**Published:** 2023-04-05

**Authors:** Bernhard Roth, Tomas Lucca Bothe, Andreas Patzak, Niklas Pilz

**Affiliations:** a Doctor’s office, Würzburg, Germany; b Institute of Translational Physiology, Charite – Universitätsmedizin Berlin, corporate member of Freie Universität Berlin, Humboldt-Universität zu Berlin, and Berlin Institute of Health, Berlin, Germany

**Keywords:** accuracy, ambulatory blood pressure monitoring, exercise, ISO protocol, validation

## Abstract

The objective of this study is to evaluate the accuracy of the oscillometric upper-arm device ABPMpro (SOMNOmedics) for ambulatory blood pressure measurement in the general population according to the Association for the Advancement of Medical Instrumentation/European Society of Hypertension/International Organization for Standardization (AAMI/ESH/ISO) Universal Standard (ISO 81060-2:2018) at rest and during dynamic exercise.

**Methods:**

Subjects were recruited to fulfill the age, sex, blood pressure (BP) and cuff distribution criteria of the AAMI/ESH/ISO standard using the same arm sequential BP measurement method. Three appropriate cuff sizes (18–24, 24–34 and 34–46 cm) of the tested device were used for the arm-varying circumferences. The inflation and deflation measurement modes of the ABPMpro were investigated.

**Results:**

For the general validation study, 100 subjects were recruited and 90 were analyzed. For validation criterion (1), the mean ± SD of the differences between ABPMpro and reference BP was 0.7 ± 7.3/–0.7 ± 5.8 mmHg (systolic/diastolic) for inflation and 1.4 ± 7.7/–0.6 ± 6.1 mmHg for deflation measurements. For criterion (2), the SD of the averaged BP differences per subject was 5.98/5.10 mmHg for inflation and 6.46/5.36 mmHg for deflation measurements, thereby passing the threshold. In the ambulatory validation study (*N* = 36), the mean difference was -1.2 ± 7.9/ 2.4 ± 6.6 mmHg for inflation and –0.7 ± 7.6/3.1 ± 7.0 mmHg for deflation measurements.

**Conclusion:**

The ABPMpro device fulfilled the ISO 81060-2:2018 requirements in the general population and in the ambulatory setting and can therefore be recommended for clinical use.

## Introduction

The study assessed the accuracy of the novel ABPMpro device (SOMNOmedics, Randersacker, Germany) developed for ambulatory blood pressure (BP) measurement (ABPM) according to the Association for the Advancement of Medical Instrumentation/European Society of Hypertension/International Organization for Standardization (AAMI/ESH/ISO) Universal Standard (ISO 81060-2:2018) in general population under clinical and ambulatory conditions.

## Methods

### Tested device

The ABPMpro is a new automated oscillometric upper-arm BP monitor developed for ABPM, with three available cuff sizes (18–24, 24–34 and 34–46 cm). The compact, tubeless device is attached directly to the cuff on the upper arm. ABPMpro has an internal activity and body position sensor enabling sleep-wake estimation. ABPMpro can be set to an inflation or deflation measurement method or, alternatively, to an inflation and deflation combinatory method. When using inflation mode, an automatic measurement during deflation will be additionally performed in case the determination of BP values during inflation failed. In the inflation/deflation combinatory method, the mean value of both methods can be displayed in the software. The manufacturer provided two identical devices that were randomly selected for the study.

### Participants

Subjects aged 12 years or older were enrolled from the Doctor’s office, Dr. Roth (Würzburg, Germany), and in an outpatient office (Dr. Roth, Würzburg, Germany). Participants were recruited to ensure that the number, sex, age, arm circumference and BP value distributions were in accordance with the AAMI/ESH/ISO universal standard [1]. Amendment 1.2020-01 was not applied.

### Validation team

The validation team consisted of a supervisor and two trained observers who are experienced in BP measurement research and were standardized to their agreement in BP measurement before study initiation [2].

### Reference blood pressure

A calibrated (accuracy according to the test protocol of the company for exactly the used device is ± 1 mmHg), standard aneroid sphygmomanometer (Boso Nova S, Jungingen, Germany), was used for simultaneous auscultatory reference BP measurements by two observers, using a dual-head teaching stethoscope (Prestige Medical, Northridge, USA). Four different cuff sizes (14–21, 22–32, 33–41, >41 cm) were selected to meet the requirements of the AAMI/ESH/ISO universal standard [1].

### Procedure

The ‘same arm sequential method’ described in the universal standard was used. A reference BP measurement was taken by two observers (R0), followed by a BP measurement with ABPMpro (T0). Validation of BP measurements [four reference auscultatory (R1–4)] alternated by three test device measurements (T1–T3) was performed. In cases of disagreement between the observers, additional pairs of measurements were generated.

Subjects in the ergometry test (older than 18 years) underwent a test protocol, which was identical to the previous one. However, the validation BP measurements were performed under dynamic exercise with a heart rate increase of 15% above the resting heart rate. The measurements were performed in the upright position with the arm supported. The cuff was at the heart level.

### Analysis

The ISO 81060-2:2018 requirements were followed diligently [1], and BP differences were calculated as the mean ± SD. For the general validation study, analysis was performed according to criteria (1) and (2), for ambulatory validation according to criterion (1). Bland–Altman plots were created to display the device-observer agreement.

### Study approval

The study was approved by the Ethics Committee of Bayerische Landesärztekammer and was registered at www.drks.de (registry number: DRKS00025866). All participants, or their parents or legal guardians, provided informed and written consent and gave written consent before the start of the study.

## Results

### General validation study

A total of 100 individuals were recruited and 90 were analyzed. Participant characteristics, BP and cuff distribution are presented in Table 1, and reasons for exclusion are in Supplementary Table S1, (Supplemental digital content 1, http://links.lww.com/BPMJ/A188). The requirements for age, sex, BP and cuff distribution were fulfilled [2]. The mean BP difference between the simultaneous observers’ measurements was −0.2 ± 0.9/0.1 ± 1.2 mmHg (SBP/DBP, range −4 to 4 mmHg). There were 50 BP readings with inter-observer disagreement >4 mmHg.

**Table 1:** Baseline characteristics for participants (*n* = 90) and validation study results (general validation study)

	Mean ± SD		Range	
Age (years)	39.7 ± 15.1		12–76	
Male/female, n (%)	47/43 (52/48)		–	
Arm circumference (cm)	29.5 ± 5.4		20–44	
Entry SBP R0 (mmHg)	126.8 ± 18		92–170	
Entry DBP R0 (mmHg)	78.3 ± 11.8		52–108	
SBP (mmHg), proportion of measurements (%)				
≤100	11			
≥140	21			
≥160	5			
DBP (mmHg), proportion of measurements (%)				
≤60	6			
≥85	21			
≥100	6			
Cuff size (cm), subjects [n (%)]				
Small (18–24)	15 (17)			
Medium (24–34)	60 (67)			
Large (34–46)	15 (17)			
	Pass requirement	Achieved		
		SBP		DBP
Criterion 1 (261 BP pairs) (inflation)				
Mean BP difference (mmHg)	≤5	0.7		–0.7
SD (mmHg)	≤8	7.3		5.8
		Pass		Pass
Criterion 2 (90 subjects)				
SD (mmHg, SBP/DBP)	≤6.90/6.90	5.98		5.10
		Pass		Pass
Result		Pass		
Criterion 1 (262 BP pairs) (deflation)				
Mean BP difference (mmHg)	≤5	1.4		–0.6
SD (mmHg)	≤8	7.7		6.1
		Pass		Pass
Criterion 2 (90 subjects)				
SD (mmHg, SBP/DBP)	≤6.80/6.91	6.46		5.36
		Pass		Pass
Result				Pass

**Fig. 1:**
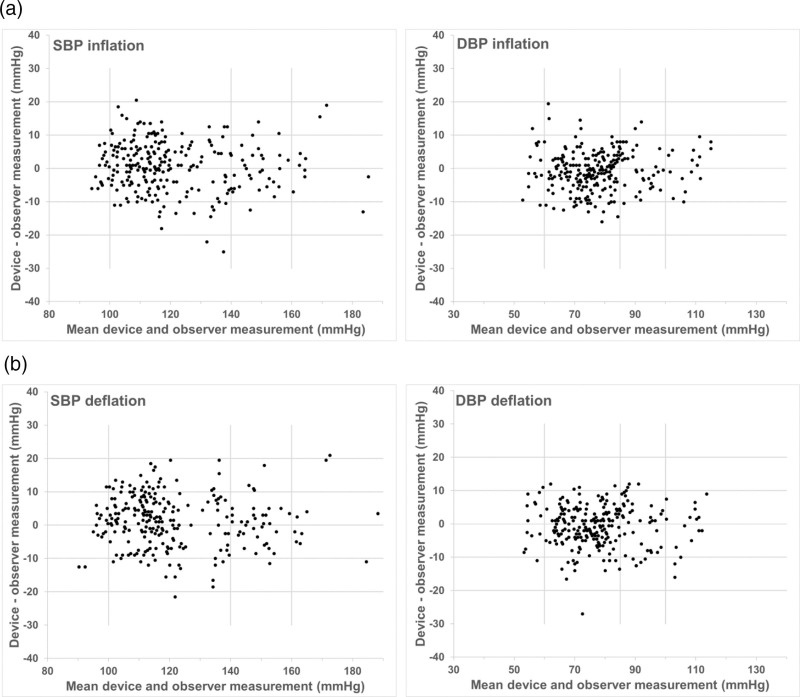
Bland–Altman scatterplots of mean ABPMpro and observer measurements for (a) inflation and (b) deflation method (general validation study).

The mean device-observer difference was 0.7 ± 7.3/−0.7 ± 5.8 mmHg (SBP/DBP) for inflation and 1.4 ± 7.7/−0.6 ± 6.1 mmHg for deflation measurements. These data are in agreement with criterion (1) (≤5 ± 8 mmHg). For criterion (2), the SD of the averaged BP differences per subject was 5.98/5.10 mmHg (SBP/DBP) for inflation and 6.46/5.36 mmHg for deflation measurements, both below the requirement. Validation criteria (1) and (2) suggest a ‘pass’ for SBP and DBP. The validation analysis is shown in Table 1, and Bland–Altman plots are shown in Fig. 1. The results of the mean calculation of the inflation and deflation measurements using the software are shown in Supplementary Table S2, Supplemental digital content 2, http://links.lww.com/BPMJ/A189 and Supplementary Figure S1, Supplemental digital content 3, http://links.lww.com/BPMJ/A190.

### Ambulatory validation study

Participant characteristics and BP distribution are presented in Table 2, reasons for exclusion in Supplementary Table S3, Supplemental digital content 4, http://links.lww.com/BPMJ/A191.

**Table 2:** Baseline characteristics for participants (*n* = 36) and validation study results (ambulatory validation study)

	Mean ± SD		Range	
Age (years)	40.9 ± 11.0		22–65	
Male/female, n (%)	20/16 (56/44)		–	
Arm circumference (cm)	30.6 ± 4.0		24–38	
Entry SBP R0 (mmHg)	127.7 ± 13.4		109–158	
Entry DBP R0 (mmHg)	81.8 ± 8.8		61–100	
SBP (mmHg), proportion of measurements (%)				
≥140	31			
	Pass requirement	Achieved		
		SBP		DBP
Criterion 1 (106 BP pairs) (inflation)				
Mean BP difference (mmHg)	≤5	–1.2		2.4
SD (mmHg)	≤8	7.9		6.6
		Pass		Pass
Criterion 1 (105 BP pairs) (deflation)				
Mean BP difference (mmHg)	≤5	–0.7		3.1
SD (mmHg)	≤8	7.6		7.0
		Pass		Pass
Result				Pass

A minimum increase in heart rate (15%) was achieved in all subjects. The average increase in heart rate was 27.3%.

**Fig. 2:**
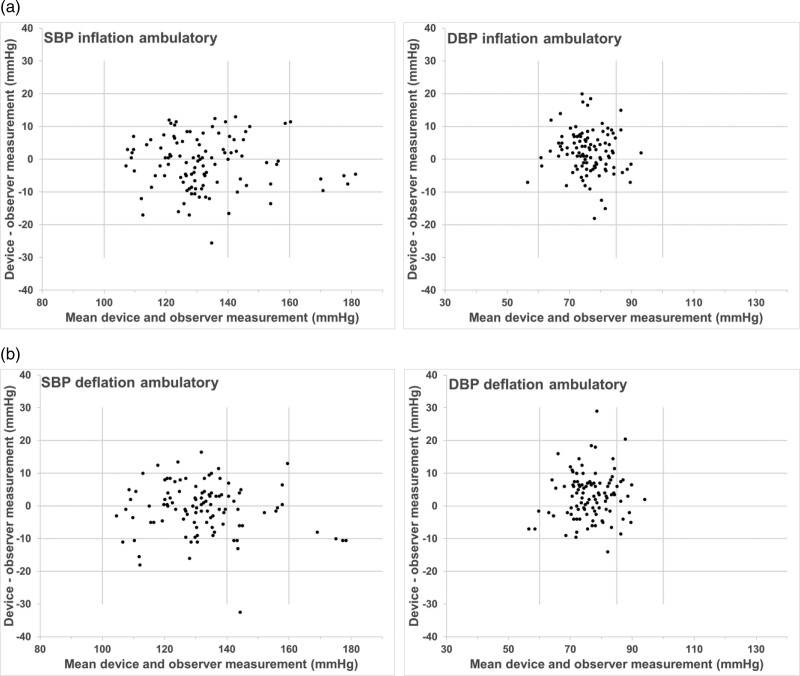
Bland–Altman plots of mean ABPMpro and observer measurements for (a) inflation and (b) deflation method (ambulatory validation study).

The mean device-observer difference was –1.2 ± 7.9/2.4 ± 6.6 mmHg (SBP/DBP) for inflation and −0.7 ± 7.6/3.1 ± 7.0 mmHg for deflation measurements. The validation analysis is shown in Table 2 and the Bland–Altman plots are shown in Fig. 2. The data agree with criterion (1) of the AAMI/ESH/ISO Universal Standard requirements. Criterion (2) is exempt for ambulatory validation studies. The results of the mean calculation of the inflation and deflation measurements using the software are shown in Supplementary Table S4, Supplemental digital content 5, http://links.lww.com/BPMJ/A192 and Supplementary Figure S2, Supplemental digital content 6, http://links.lww.com/BPMJ/A193.

## Discussion

The ABPMpro provided reliable results for SBP and DBP values, regardless of the BP range. An innovative aspect of the ABPMpro is the ability to measure BP during the inflation or deflation process. This novel feature allows deflation measurement to be performed as a backup if the measurement is affected during inflation. The ABPMpro achieved criteria (1) and (2) of the AAMI/ESH/ISO Universal Standard (ISO 81060-2:2018) at rest using either the inflation or deflation measurement technique as well as software calculation of mean values.

A limitation of the study is that ISO Amendment 1.2020-01 of the standard was not considered when planning and conducting the study. We recruited only 7.9% of the required 20% patients with arm circumferences over 39 cm. Therefore, the performance of the system is not validated for this group of patients.

The AAMI/ESH/ISO Universal Standard (ISO 81060-2:2018) describes a procedure for validating ambulatory devices. Therefore, measurements must be performed under elevated heart rate conditions. Data from the ambulatory validation study show that the ABPMpro fulfills criterion (1) for SBP and DBP in either the inflation or deflation method, as well as by calculating the mean values using the software.

Concluding, this study shows that the ABPMpro fulfills the accuracy criteria of the AAMI/ESH/ISO Universal Standard (ISO 81060-2:2018), both in the general validation as well as in the ambulatory validation study. Thus, the device is recommended for clinical use in the general population.

## Acknowledgements

The devices were supplied by SOMNOmedics GmbH, which also provided funding to B.R. for the study.

The device is not yet approved by the FDA.

### Conflicts of interest

T.L.B. and A.P. advise SOMNOmedics on blood pressure measurement and received travel support. For the remaining authors, there are no conflicts of interest.

## Supplementary Material












