# Supplementary material for: Validation of the ABPMpro ambulatory blood pressure monitor in the general population according to AAMI/ESH/ISO Universal Standard (ISO 81060-2:2018)
Source: Blood Press Monit. 2023 Apr 5;28(3):158–62. doi: 10.1097/MBP.0000000000000640 (PMC10132455; doi:10.1097/MBP.0000000000000640)
Supplement: Supplementary file 1 [file bpmj-28-158-s001.pdf]

Table S 1: Participants recruited and excluded from the analyses in the general validation study

	Subjects
Recruited	100
Excluded	10
Reasons for exclusion	
- reference BP variability (>12/8 mmHg for systolic/diastolic)	6
- Korotkoff sounds not audible	1
- arrhythmia	1
- exclusion criteria overlooked	1
- range adjustment	1
Analysed	90
