# Supplementary material for: Validation of the ABPMpro ambulatory blood pressure monitor in the general population according to AAMI/ESH/ISO Universal Standard (ISO 81060-2:2018)
Source: Blood Press Monit. 2023 Apr 5;28(3):158–62. doi: 10.1097/MBP.0000000000000640 (PMC10132455; doi:10.1097/MBP.0000000000000640)
Supplement: Supplementary file 2 [file bpmj-28-158-s002.pdf]

Table S 2: Validation study results (general validation study), for mean calculation of inflation and deflation measurements.

	Pass requirement	***Achieved***	
		SBP	***DBP***
**Criterion 1** *(262 BP pairs) (mean inflation and deflation)*			
Mean BP difference (mmHg)	≤ 5	1.1	-0.7
SD (mmHg)	≤ 8	6.9	5.5
		Pass	*Pass*
**Criterion 2** *(90 subjects)*			
SD (mmHg, SBP / DBP)	≤ 6.9 / 6.9	5.98	4.97
		Pass	*Pass*
**Result**		***Pass***	
