# Supplementary material for: Validation of the ABPMpro ambulatory blood pressure monitor in the general population according to AAMI/ESH/ISO Universal Standard (ISO 81060-2:2018)
Source: Blood Press Monit. 2023 Apr 5;28(3):158–62. doi: 10.1097/MBP.0000000000000640 (PMC10132455; doi:10.1097/MBP.0000000000000640)

**Figure S 1:** Bland-Altman scatterplots of mean ABPMpro (mean calculation of inflation and deflation) and observer measurements for SBP (left) and DBP (right) (general validation study).

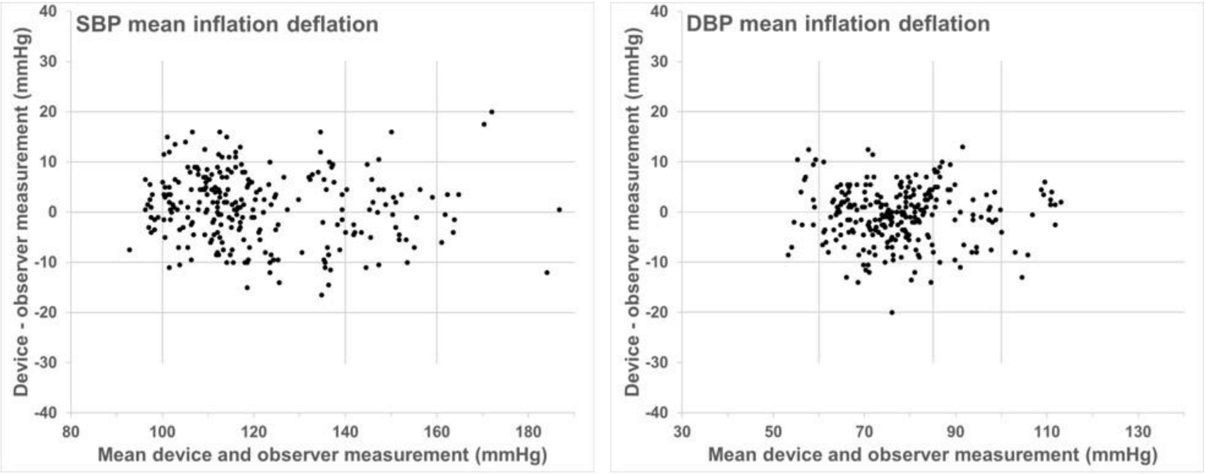

Supplement: Supplementary file 3 [file bpmj-28-158-s003.pdf]
