# Supplementary material for: Validation of the ABPMpro ambulatory blood pressure monitor in the general population according to AAMI/ESH/ISO Universal Standard (ISO 81060-2:2018)
Source: Blood Press Monit. 2023 Apr 5;28(3):158–62. doi: 10.1097/MBP.0000000000000640 (PMC10132455; doi:10.1097/MBP.0000000000000640)
Supplement: Supplementary file 4 [file bpmj-28-158-s004.pdf]

Table S 3: Participants recruited and excluded from the analysis in the ambulatory validation study.

	Subjects
Recruited	58
Excluded	22
Reasons for exclusion	
- Korotkoff sounds not audible	3
- Participant noncompliance (e.g., movement)	17
- insufficient HR rise	1
- termination at the request of the subject	1
Analysed	36
