# Supplementary material for: Validation of the ABPMpro ambulatory blood pressure monitor in the general population according to AAMI/ESH/ISO Universal Standard (ISO 81060-2:2018)
Source: Blood Press Monit. 2023 Apr 5;28(3):158–62. doi: 10.1097/MBP.0000000000000640 (PMC10132455; doi:10.1097/MBP.0000000000000640)

Figure S 1: Bland-Altman plots of mean ABPMpro (mean calculation of inflation and deflation) and observer measurements for SBP (left) and DBP (right) (ambulatory validation study).

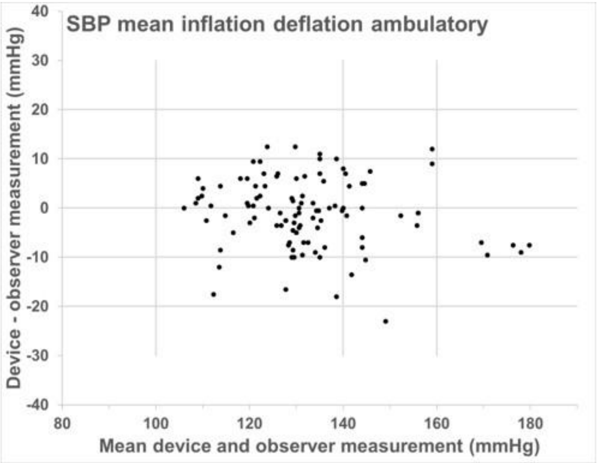

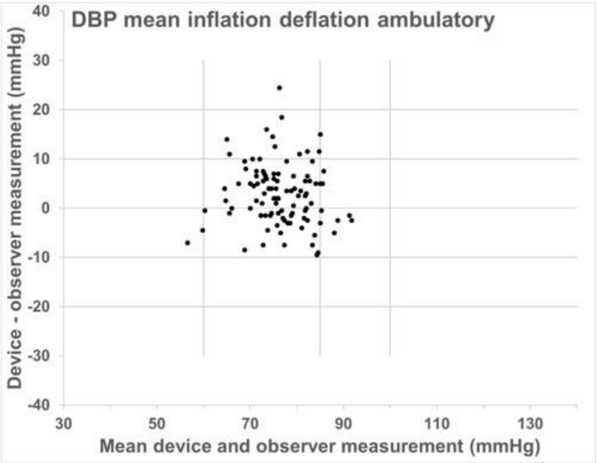

Supplement: Supplementary file 6 [file bpmj-28-158-s006.pdf]
